# Supplementary material for: Integrated multi-omics analysis identifies microbial and metabolic signatures and drivers of CNS autoimmunity
Source: mSystems. 2026 Aug 11;11(9):e00002-26. doi: 10.1128/msystems.00002-26 (PMC13595924; doi:10.1128/msystems.00002-26)
Supplement: Supplementary file 1 [file reviewer-comments.pdf]

ASM | mSystems

Integrated multi-omics analysis identifies microbial and metabolic signatures and drivers of CNS autoimmunity

Theresa Montgomery, Emily Nelson, Lauren Downs, Eamonn Heney, Margaret Frances Lee, Cameron Martino, Daniel McDonald, Gibraan Rahman, Rob Knight, and Dimitry Krementsov

Corresponding Author(s): Theresa Montgomery, University of Vermont College of Nursing and Health Sciences

Review Timeline:

Submission Date:	January 6, 2026
Editorial Decision:	April 16, 2026
Revision Received:	June 29, 2026
Accepted:	July 15, 2026

Editor: Emily Cope

Reviewer(s): The reviewers have opted to remain anonymous.

Transaction Report:

(Note: With the exception of the correction of typographical or spelling errors that could be a source of ambiguity, letters and reports are not edited. The original formatting of letters and referee reports may not be reflected in this compilation.)

DOI: https://doi.org/10.1128/msystems.00002-26

Re: mSystems00002-26 (***Integrated multi-omics analysis identifies microbial and metabolic signatures and drivers of CNS autoimmunity***)

Dear Dr. Theresa Lynn Montgomery:

Thank you for the privilege of reviewing your work and for your patience as we reviewed your manuscript. Below you will find my comments, instructions from the mSystems editorial office, and the reviewer comments.

Please return the manuscript within 60 days; if you cannot complete the modification within this time period, please contact me. If you do not wish to modify the manuscript and prefer to submit it to another journal, notify me immediately so that the manuscript may be formally withdrawn from consideration by mSystems.

Revision Guidelines
To submit your modified manuscript, log into the submission site at https://msystems.msubmit.net/cgi-bin/main.plex. Go to Author Tasks and click the appropriate manuscript title to begin. The information you entered when you first submitted the paper will be displayed; update this as necessary. Note the following requirements:

• Upload point-by-point responses to the issues raised by the reviewers in a file named "Response to Reviewers," NOT in your cover letter.
• Upload a compare copy of the manuscript (without figures) as a "Marked-Up Manuscript" file.
• Upload a clean .DOC/.DOCX version of the revised manuscript and remove the previous version.
• Each figure must be uploaded as a separate, editable, high-resolution file (TIFF or EPS preferred), and any multipanel figures must be assembled into one file.
• Any supplemental material intended for posting by ASM should be uploaded with their legends separate from the main manuscript. You can combine all supplemental material into one file (preferred) or split it into a maximum of 10 files with all associated legends included.

For complete guidelines on revision requirements, see our Submission and Review Process webpage. Submission of a paper that does not conform to guidelines may delay acceptance of your manuscript.

Data availability: ASM policy requires that data be available to the public upon online posting of the article, so please verify all links to sequence records, if present, and make sure that each number retrieves the full record of the data. If a new accession number is not linked or a link is broken, provide mSystems production staff with the correct URL for the record. If the accession numbers for new data are not publicly accessible before the expected online posting of the article, publication may be delayed; please contact production staff (mSystems@asmusa.org) immediately with the expected release date.

Publication Fees: For information on publication fees and which article types are subject to charges, visit our website. If your manuscript is accepted for publication and any fees apply, you will be contacted separately about payment during the production process; please follow the instructions in that e-mail. Arrangements for payment must be made before your article is published.

ASM Membership: Corresponding authors may join or renew ASM membership to obtain discounts on publication fees. Need to upgrade your membership level? Please contact Customer Service at Service@asmusa.org.

The ASM Journals program strives for constant improvement in our submission and publication process. Please tell us how we can improve your experience by taking this quick Author Survey.

Thank you for submitting your paper to mSystems.

Sincerely,
Emily Cope
Editor
mSystems

Reviewer #1 (Comments for the Author):

In the manuscript from Montgomery et al, the authors investigate diet-microbe-metabolomic interactions linked to CNS autoimmunity. Specifically, they study longitudinal microbiome and metabolomic changes associated with EAE severity in mice colonized with L. reuteri+ microbiota (vs. not) given a high vs. low trypotphan diet. Following data analysis, they confirm the role of two microbial metabolites and demonstrate they worsen EAE, spinal cord inflammation, as well as brain inflammation. Major

strengths of the study are the combination of multi-omics approaches and two different causal studies (diet-microbe and metabolite). This manuscript adds to the field of microbe-diet-metabolite drivers of neurologic diseases. I have minor comments below.

1. Line 175 -178. The exploratory analysis states that L. reuteri increased Akkermansia and Turicibacter at the expense of Faecalibacterium, but these patterns doe no appear consistently in all mice. Better to cut these lines and focus on data supported by the statistical analysis in the following lines (179-203). The same comment applies to lines 231-235

2. Figure 2D and Figure 3D. Visualizing the top 11 taxa out of 100% may distort the data. Better to plot the actual relative abundances, rather than recalculating the proportion. For fig. 3E, Change "B6 decreased" to either "B6 + L reuteri decreased" or "B6 increased" to align with other labels.

3. For context, it would be helpful to reproduce the EAE scores from the original studies. This is often possible when writing and obtaining permission for reproduction and citing the article in the new paper / figure legend.

4. Figure 5 A and B. Recommend to add the genus abbreviation. Eg. "F". rodentium instead of rodentium.

5. Lines 430-433, and line 626 - For the microbes that were associated with EAE, were they increased or decreased in mice with more severe disease? What is the direction of the association? Anaertruncus has been shown to ameliorate eae.

6. Fig 5 and 6. The text is small in some of the figures. In figure 5B, there appear to be more rows and columns than microbial names and metabolites. Are there replicates displayed here?

7. lines 650 -686. This paragraph is more than a page. I recommend to break this up.

8. Do the metabolites also change the gut microbiota? Is this a potential secondary mechanism by which they influence EAE?

Reviewer #2 (Comments for the Author):

This is an exciting manuscript that builds on the author's prior findings that Limosilactobacillus reuteri exacerbates MS in a tryptophan dependent manner in a mouse model (EAE). They performed a longitudinal study of L reuteri in the EAE model on high and low tryptophan diets. They found that under long term dietary tryptophan exposure and EAE progression, L. reuteri elicited profound effects on the gut microbiome and metabolome. Metabolomic features that were strongly associated with EAE severity exacerbated EAE. The integrated analysis of microbiome and metabolomic data in a longitudinal study of EAE and diet are a strength. I have only minor comments below:

When reporting p values in the results text, it would be helpful to include the statistical test used to generate the p value

The text size is very small, especially in the volcano plots, limiting the interpretability. I'd recommend reducing the number of features highlighted in the plot itself with text. A supplemental table could be generated to describe all significant features. These do appear to be included in the supplemental information (e.g., Table S3-S7?) but these aren't referenced in the Results text.

Similar comment above related to Figure 5. The bi-plot is difficult to interpret because of the dense and small text size. This is currently presented as a single multi-panel figure but I wonder if it could be broken up into two figures to allow expansion of the networks presented in D and E. These are almost impossible to read/interpret.

Figure 7 - need to clarify the meaning of features highlighted in blue v. red v. not highlighted

A more robust discussion of the limitations of the study could be included

How many mice were housed per cage and across experimental conditions? Cage effect is not discussed or analyzed as a confounder

We thank the reviewers for their thorough and constructive review of our manuscript. Below we provide a point-by-point response to their specific concerns. Changes in the revised manuscript are tracked by highlighting within the compare copy of the manuscript.

Reviewer #1 (Comments for the Author):

1. Line 175 -178. The exploratory analysis states that L. reuteri increased Akkermansia and Turicibacter at the expense of Faecalibacterium, but these patterns do no appear consistently in all mice. Better to cut these lines and focus on data supported by the statistical analysis in the following lines (179-203). The same comment applies to lines 231-235

To substantiate these exploratory claims, we performed statistical testing at the genus level (new Fig. S2A and S2B) and found that the increase in Akkermansia and Turicibacter but not Faecalibacterium was significant at the 1 week time point. We also performed the same analysis at the 5 week time (Fig. S2B) an similarly modified our claims. We have revised the results section (lines 175 and lines 218-219) in accordance.

2. Figure 2D and Figure 3D. Visualizing the top 11 taxa out of 100% may distort the data. Better to plot the actual relative abundances, rather than recalculating the proportion. For fig. 3E, Change "B6 decreased" to either "B6 + L reuteri decreased" or "B6 increased" to align with other labels.

We agree that compositional distortion can arise when subsets of taxa are re-normalized. To further address this concern, we have included new supplementary Figure S3, which presents the same data using the full relative abundances without re-scaling. Our intent with the visualization in the main figures is to provide a concise summary of the dominant genera, while still accounting for low-abundance taxa through inclusion of an aggregated “Others” category. Importantly, all underlying analyses were conducted on the full relative abundance dataset prior to any aggregation, and no statistical conclusions were based on the subset visualization.

We have corrected the label in Fig. 3E.

3. For context, it would be helpful to reproduce the EAE scores from the original studies. This is often possible when writing and obtaining permission for reproduction and citing the article in the new paper / figure legend.

We have included new Fig. S1A-F to reproduce EAE scores and provide additional parameters of disease severity. We have expanded our description of this finding on lines 144-147.

4. Figure 5 A and B. Recommend to add the genus abbreviation. Eg. "F". rodentium instead of rodentium.

Genus abbreviations have been added accordingly.

5. Lines 430-433, and line 626 - For the microbes that were associated with EAE, were they increased or decreased in mice with more severe disease? What is the direction of the association? Anaertruncus has been shown to ameliorate eae.

We appreciate this suggestion, this will provide more insight for the readers. We have added Fig. S4 to delineate the directionality of features associated with EAE disease severity. Specifically, metabolomic and microbiome features were analyzed using LinDA to identify those that significantly correlate with cumulative disease score (CDS). Results are presented as log2 fold-change per unit increase in CDS, where positive values indicate higher feature abundance with increasing disease severity, and negative values indicate lower abundance with increasing disease severity.

This analysis revealed that the Anaerotruncus ASV is in fact associated with increased disease severity, which is in fact consistent with the prior report, although surprisingly that report indeed found the opposing result (disease suppression) when directly treating with this bacterium. These findings are presented in the results section lines 373-375 and 387-389 and discussed in the discussion on lines 525-532.

6. Fig 5 and 6. The text is small in some of the figures. In figure 5B, there appear to be more rows and columns than microbial names and metabolites. Are there replicates displayed here?

We have increased text size in figures 5 and 6 where possible, as suggested. In figure 5B, not all metabolites and ASVs are labeled intentionally, for legibility of the text – we have indicated this in the figure legend for transparency. We have also added Table S22: ASV to Metabolite Correlation Matrix from jRPCA, which contains the full dataset depicted in the heatmap with all ASVs and metabolites for the reader's reference. Importantly, all downstream analyses are reflective of this total dataset.

We are unsure what type of replicates the reviewer is referring to, but the matrix represents aggregate correlations derived from the entire set of animals (i.e. biological replicates). The rows and columns all represent individual unique metabolites or ASVs, and they are not duplicated/replicated or split out/grouped by sample. We hope that Table S22 makes this apparent.

7. lines 650 -686. This paragraph is more than a page. I recommend to break this up.

We have broken this into two paragraphs, as suggested.

8. Do the metabolites also change the gut microbiota? Is this a potential secondary mechanism by which they influence EAE?

This is an intriguing question that warrants discussion and future investigation. There is substantial evidence that both circulating and gut luminal metabolites can shape gut microbial composition and function. These effects may occur through direct interactions with microbial communities, as well as indirectly via modulation of host processes such as immune responses and epithelial barrier integrity. Accordingly, it is plausible that metabolite-driven alterations in the microbiota could represent an additional, secondary mechanism contributing to their effects on EAE. We have strengthened our discussion of this indirect mechanistic relationship on lines 536-539.

Reviewer #2 (Comments for the Author):

When reporting p values in the results text, it would be helpful to include the statistical test used to generate the p value

Statistical tests have been indicated, as suggested - including a description of LINDA on lines 178-179.

The text size is very small, especially in the volcano plots, limiting the interpretability. I'd recommend reducing the number of features highlighted in the plot itself with text. A supplemental table could be generated to describe all significant features. These do appear to be included in the supplemental information (e.g., Table S3-S7?) but these aren't referenced in the Results text.

We have increased font sizes in all volcano plots, reduced the number of features, and referenced the supplemental tables throughout, as suggested.

Similar comment above related to Figure 5. The bi-plot is difficult to interpret because of the dense and small text size. This is currently presented as a single multi-panel figure but I wonder if it could be broken up into two figures to allow expansion of the networks presented in D and E. These are almost impossible to read/interpret.

We have increased font size in Figure 5.

Figure 7 - need to clarify the meaning of features highlighted in blue v. red v. not highlighted

We agree the red and blue highlighted text in figure 7 was confusing and have removed it.

A more robust discussion of the limitations of the study could be included

We have included a study limitations section on line 625-648.

How many mice were housed per cage and across experimental conditions? Cage effect is not discussed or analyzed as a confounder

Experimental microbiomes are vertically transmitted from 2 parental breeding pairs per microbiome context (B6 and B6 + *L. reuteri*). Resulting offspring are weaned in cages of 4-5 and later randomized to experimental diets (low or high tryptophan) at ~7 weeks of age in 2-3 mice per cage to control for parental and cage effect between microbiomes and diets. We have added the number of mice per cages to the methods section on line 694. Because of the randomization strategy – initially co-housed mice are randomized to two different diets and different cages, we feel that the cage effect was not a major confounder when analyzing effect of diet on microbiome. Furthermore, we included analysis of at least 2 different cages per treatment group, thus minimizing potential outlier/drift effects of a single cage. This information has been added to the materials and methods.

Re: mSystems00002-26R1 (***Integrated multi-omics analysis identifies microbial and metabolic signatures and drivers of CNS autoimmunity***)

Dear Dr. Theresa Lynn Montgomery:

Your manuscript has been accepted, and I am forwarding it to the ASM production staff for publication. Your paper will first be checked to make sure all elements meet the technical requirements. ASM staff will contact you if anything needs to be revised before copyediting and production can begin. Otherwise, you will be notified when your proofs are ready to be viewed.

Data Availability: ASM policy requires that data be available to the public upon online posting of the article, so please verify all links to sequence records, if present, and make sure that each number retrieves the full record of the data. If a new accession number is not linked or a link is broken, provide production staff with the correct URL for the record. If the accession numbers for new data are not publicly accessible before the expected online posting of the article, publication may be delayed; please contact ASM production staff immediately with the expected release date.

Publication Fees: For information on publication fees and which article types have charges, please visit our website. We have partnered with Copyright Clearance Center (CCC) to collect author charges. If fees apply to your paper, you will receive a message from no-reply@copyright.com with further instructions. For questions related to paying charges through RightsLink, please contact CCC at ASM_Support@copyright.com or toll free at +1-877-622-5543. CCC makes every attempt to respond to all emails within 24 hours.

ASM Membership: Corresponding authors may join or renew ASM membership to obtain discounts on publication fees. Need to upgrade your membership level? Please contact Customer Service at Service@asmusa.org.

Embargo Policy: A press release may be issued as soon as the manuscript is posted on the mSystems Latest Articles webpage. The corresponding author will receive an email with the subject line "ASM Journals Author Services Notification" when the article is available online.

Cover Image Submissions: If you would like to submit a potential Cover Image, please email a file and a short legend to msystems@asmusa.org. Please note that we can only consider images that (i) the authors created or own and (ii) have not been previously published. By submitting, you agree that the image can be used under the same terms as the published article. Image File requirements: TIF/EPS, 7.5 inches wide by 8.25 inches tall (at least 2,250 pixels wide by 2,475 pixels tall), minimum 300 dpi resolution (600 dpi preferred), RGB, and no figure elements, e.g., arrows or panel labels. The legend should be a short description of the image, 1-2 sentences recommended. Please download and use this interactive template in Adobe to ensure that your proposed cover image meets our size requirements (https://journals.asm.org/pb-assets/pdf-text-excel-files/ASM-Interactive-Sizing-Cover-Template-1715689791.pdf).

Thank you for submitting your paper to mSystems.

Sincerely,
Emily Cope
Editor
mSystems
