# Supplementary material for: Integrated multi-omics analysis identifies microbial and metabolic signatures and drivers of CNS autoimmunity
Source: mSystems. 2026 Aug 11;11(9):e00002-26. doi: 10.1128/msystems.00002-26 (PMC13595924; doi:10.1128/msystems.00002-26)
Supplement: Supplementary file 2 [file msystems.00002-26-s0001.docx]

**Figure S1. *L. reuteri* drives CNS autoimmunity in a dietary tryptophan-dependent manner.** EAE was evaluated in mice colonized with a B6 or B6 + *L. reuteri* gut microbiome, randomized to a high (0.8%) or low (0.02%) tryptophan diet 1 week prior to EAE induction as shown in the schematic in **Fig. 1** and as previously published [59]. EAE disease courses reflected as mean daily clinical score, with overall significance determined by Friedman's non-parametric two-way ANOVA in high (**A**) and low (**B**) tryptophan fed mice. EAE quantifiable traits including cumulative disease score (**C**), severity index (D), disease incidence (**E**), number of days affected (**F**), day of disease onset (**G**), and peak disease score (**H**) with significant difference determined using one-way ANOVA with a Sidak’s post hoc comparisons test or chi-square test for disease incidence.

**Figure S2. *L. reuteri* colonization significantly alters gut microbiome composition.** Differentially abundant genera 1 week (**A**) and 5 weeks (**B**) post dietary intervention between the B6 and B6 + *L. reuteri* gut microbiomes as determined by LinDA when corrected for diet, using a Benjamini–Hochberg padj ≤ 0.05 cutoff.

**Figure S3. Relative abundance of bacterial genera.** Stacked bar plots depicting the relative abundance of the top 11 most abundant genera, expressed as a fraction of total sequencing reads (without re-scaling to 100%), at 1 week (**A**) and 5 week (**B**) post dietary intervention in the B6 and B6 + *L. reuteri* gut microbiomes.

**Figure S4. Directionality of microbiome and metabolite associations with EAE clinical disease severity (CDS).** Features identified by random forest regression as predictive of EAE were evaluated using LinDA to determine the direction of association with clinical disease severity. LinDA models the association between feature abundance and CDS as a continuous variable, where the coefficient represents the change in abundance per one-unit increase in CDS, allowing determination of whether features are enriched or depleted with increasing disease severity. Shown are bacterial ASVs (**A**) and metabolites (**B**) significantly associated with CDS (padj ≤ 0.05), with positive coefficients indicating enrichment and negative coefficients indicating depletion in mice with more severe disease.

**Figure S5. Identification of a colony of C57BL/6J mice that are naturally devoid of *L. reuteri* and *L. murinus*. (A)** Relative abundance of *L. reuteri* and *L. murinus* in C57BL/6J mice from room AX29 distributed by the Jackson Laboratory was determined by species-specific qPCR and normalized by eubacterial pan-specific qPCR abundance. Historical B6 and B6 + *L. reuteri* abundance data was included as species-specific positive controls. (**B**) Water intake per cage of 5 mice. (**C**) Cage dosing in mg of metabolite per kg body weight. (**D**) Average experimental dosing per mouse in mg of metabolite per kg body weight. Water bottles and mouse weights were taken every other day.
